# Synthesis and Structural Characterization of 1-[2-(5-Nitro-1*H*-indol-2-yl)phenyl]methylpyridinium Chloride

**DOI:** 10.3390/molecules16097627

**Published:** 2011-09-06

**Authors:** John B. Bremner, Siritron Samosorn, Brian W. Skelton, Allan H. White

**Affiliations:** 1 School of Chemistry, University of Wollongong, Wollongong, NSW 2522, Australia; 2 Department of Chemistry, Srinakharinwirot University, Bangkok 10110, Thailand; 3 School of Biomedical, Biomolecular and Chemical Sciences, Chemistry M313, The University of Western Australia, Crawley, W.A. 6009, Australia

**Keywords:** indole, pyridinium salt, X-ray crystal structure

## Abstract

In the course of studies on hybrid antibacterials incorporating 2-aryl-5-nitro-1*H*-indole moieties as potential bacterial NorA efflux pump inhibitors, the compound 1-[2-(5-nitro-1*H*-indol-2-yl)phenyl]methylpyridinium chloride (**2**) was synthesized and structurally characterized. This pyridinium chloride salt crystallized in the monoclinic space group *P*2_1_/*c* with the following unit cell dimensions: *a* 10.274(3) Å, *b* 13.101(4) Å, *c* 13.439(4) Å, β 107.702(7)°, *V* 1723.2(9) Å^3^, *Z* (f.u.) = 4; *R*1 = 0.048, and *wR*2 = 0.13. Of interest in the single crystal X-ray structure is the (intramolecular) disposition of the pyridinium plane over the indole heterocyclic residue [interplanar dihedral angle 17.91(4)°].

## 1. Introduction

Quaternary pyridinium salts are of continuing interest as biocidal agents [1,2,3,4] and we have synthesised one such salt as part of a broader project on new hybrid antibacterial agents incorporating 2-aryl-5-nitro-1*H*-indole moieties as bacterial NorA efflux pump inhibitors [5,6,7,8,9]. This pump in the human bacterial pathogen, *Staphylococcus aureus*, is known to compromise the activity of a range of antibacterials including biocidal quaternary salts and the quaternary alkaloid salt berberine [10]. In an attempt to introduce an *N*-tosyl protecting group into the nitro indolic pump inhibitor **1** [5], reaction with *p*-toluenesulfonyl chloride in the presence of pyridine as a base was investigated. This resulted, however, in the novel substituted pyridinium chloride salt **2**, which was a potential hybrid antibacterial with an antibacterial benzylic pyridinium group (and potential NorA pump substrate) tethered to a NorA efflux pump blocking component. The synthesis and characterization of **2**, together with a single crystal X-ray structure determination, is now reported in this paper.

## 2. Results and Discussion

**Scheme 1:**
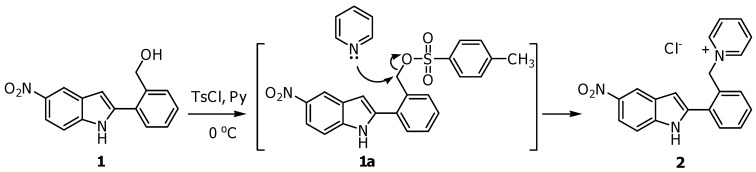
Synthesis of the pyridinium salt **2**.

The pyridinium salt **2** was synthesised from the previously described [5] indole alcohol **1** upon reaction with p-toluenesulfonyl chloride in the presence of pyridine followed by work up of the reaction mixture with exposure to hydrochloric acid (Scheme 1). The salt **2** was isolated in moderate yield and was characterized through spectroscopic data. The absence of any evidence for the presence of an aromatic methyl group (for the *p*-toluenesulfonyl functionality) was clear from the ^1^H- and ^13^C-NMR data, while the incorporation of a pyridinium moiety was indicated from the characteristic low field signals in the aromatic region of the ^1^H- and ^13^C-NMR spectra. Peak assignments in the latter spectrum were confirmed, where possible, through HMBC and HSQC experiments.

The synthesis of **2** presumably proceeds through the intermediate *p*-toluenesulfonate derivative **1a**, followed by nucleophilic displacement with pyridine [11] and then sulfonate/chloride exchange on the addition of concentrated HCl in the reaction work up (Scheme 1). The nucleophilic displacement would be expected to be facile at the benzylic site which is also activated through extended N-lone pair electron delocalisation.

**Figure 1:**
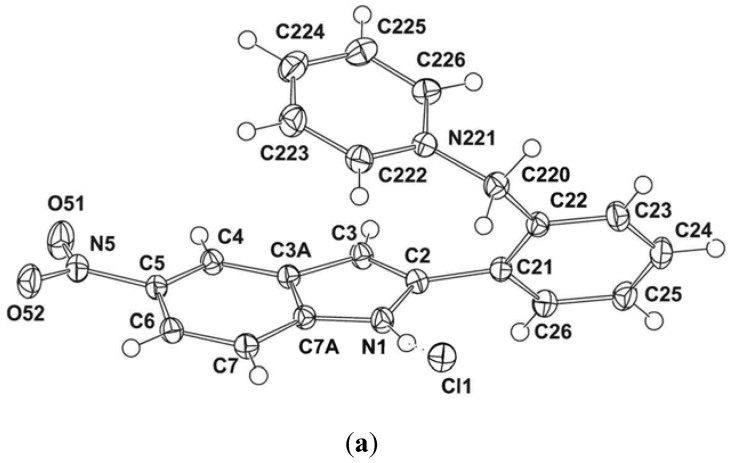

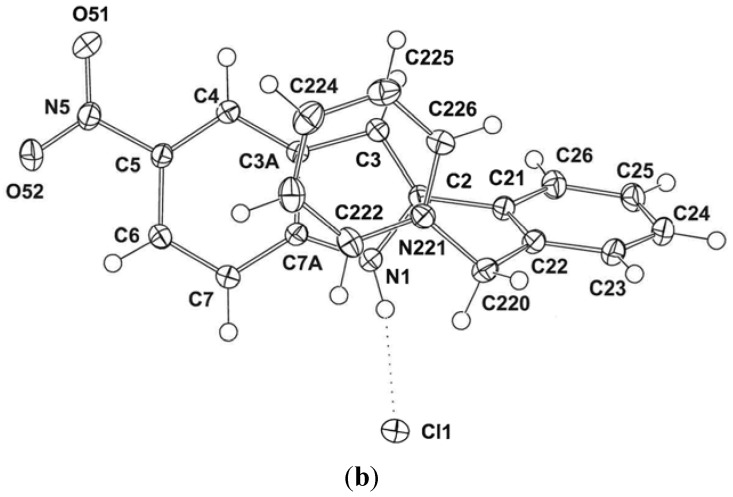
Projection of the ion-pair of the pyridinium chloride salt **2** (**a**) through and (**b**) normal to the plane of the indole component, showing crystallographic numbering, and 50% probability amplitude displacement ellipsoids for the non-hydrogen atoms, with hydrogen atoms having arbitrary radii of 0.1 Å. Crystallographic numbering used is shown.

A single crystal X-ray study provided unequivocal structural confirmation for **2** including the presence of the chloride counterion (Table 1; Figure 1a). The compound is ionic and a single formula unit devoid of crystallographic symmetry comprises the asymmetric unit of the structure. The determination is nicely precise with non-hydrogen geometries being summarized in Table 2. The quaternary cation and its stereochemistry is depicted in Figure 1a, together with the associated chloride ion. Interestingly, the pyridinium ring plane lies over that of the heterocyclic component of the indole, C(2)…N(221) being only 2.930(2) Å (Figure 1b) possibly as a result of a favourable electronic interaction between the electron deficient pyridinium moiety and the electron rich fused pyrrolo ring in the indole; the other interatomic distances are more divergent with the two ring systems having an interplanar dihedral angle of 17.91(4)°. The substituent pattern introduces some irregular features into the C6 ring of the indole fragment: C(6)-C(7) is short (1.3749(15) Å) while C(3a)-C(7a) is long (1.4217(14) Å) with the angles at the opposed C(5,7a) enlarged (124.14(10), 122.70(9)°) and those at C(4,7) diminished (116.93(9), 117.69(9)°). At C(2) there is a gross asymmetry of *ca.* 10° in the exocyclic angles, despite the twist of the pendant aromatic ring plane (Table 2). The angle sum at the pyridinium nitrogen atom is 360._0_°. The chloride ion has only one close contact: Cl...N,H(1) 3.0937(12), 2.28 Å (Cl…H(1)-N 154°), and is sufficiently close to the positive charge to be considered associated with the quaternized pyridinium nitrogen atom (Figure 1a), so that it resides in the lattice as an ion-pair. Crystal packing is dominated by inversion-related interactions between the planar residues, and diverse interspecies hydrogen contacts to other nitro and chloride components.

A preliminary investigation of the antibacterial properties of compound **2** indicated it had only weak antibacterial activity against *Staphylococcus aureus* (K2361 strain overexpressing the NorA pump) and low pump inhibitory activity with respect to the antibacterial berberine in this mutant strain. In view of this weak activity, the synthesis of other related quaternary salt analogues was not pursued, although such analogues should be readily accessible *via* the same general route.

## 3. Experimental

### 3.1. General

The reaction was monitored by thin-layer chromatography (TLC) on Merck Silica gel 60 F_254_ with a thickness of 0.2 mm on an aluminium sheet, and the compounds were detected by examination under ultraviolet light and by exposure to iodine vapour. High resolution electron impact (EI+) MS was run using a VG Autospec spectrometer operating at 70 eV and a source temperature of 250 °C with PFK reference. The ^1^H- and ^13^C-NMR were determined at 299.92 and 75.42 MHz, respectively, with a Varian Unity-300 spectrometer. The melting point (mp) determination was recorded on a Reichert melting point apparatus and is reported uncorrected.

### 3.2. Synthesis of 1-[2-(5-Nitro-1H-indol-2-yl)phenyl]methylpyridinium Chloride *(**2**)*

To a solution of indole alcohol **1** (283.3 mg, 1.05 mmol) in dry pyridine (3 mL) was added *p*-toluenesulfonyl chloride (294.0 mg, 1.5 mmol) at 0 °C, and the mixture then stirred for 20 h. The resulting yellow suspension was poured into a mixture of 32% HCl (1.5 mL) in crushed ice (5 g), then filtered, and washed with H_2_O then Et_2_O. The yellow solid was dried to yield the indole pyridinium salt **2** (217.1 mg, 65.8%). Recrystallization from MeOH and DCM afforded the salt **2** as yellow prisms (44.3 mg), m.p. 231 °C (decomp.). ^1^H-NMR (CD_3_OD) δ 6.01 (s, 2H, CH_2_), 6.68 (s, 1H, H-3), 7.49 (br.d, *J* = 9 Hz, 1H, H-7), 7.58–7.64 (m, 4H, H-3′, H-4′, H-5′, H-6′ in the 2-aryl group), 7.82–7.87 (m, 2H, H-3″, H-5″ in the pyridine ring), 8.07 (dd, *J* = 9.1, 2.2 Hz, 1H, H-6), 8.40–8.50 (m, 1H, H-4″), 8.54 (dd, *J* = 2.2, 0.4 Hz, 1H, H-4), 8.60 (dd, *J* = 6.9, 1.2 Hz, 2H, H-2″, H-6″). ^13^C-NMR (CD_3_OD) δ 64.2 (CH_2_), 105.7 (C-3), 112.6 (C-7), 118.5 (C-6), 118.6 (C-4), 129.0 (C-3a), 129.1 (3C, ArCH), 131.1 (ArCH), 131.5 (ArCH), 132.1 (ArCH), 132.5 (ArCH), 133.1 (C-2), 134.1 (C-2′), 139.9 (C-7a), 141.1 (C-1′), 143.1 (C-5), 145.9 (ArCH), 147.1 (ArCH). HRMS (EI): *m/z* calcd for C_20_H_16_N_3_O_2_ [Quaternary ion]: 330.1243; found: 330.1251.

### 3.3. X-ray Structure Determination

A full sphere of CCD area-detector diffractometer data was measured on a pale-yellow prism (Bruker AXS instrument; *ω*-scans, 2θ_max_ = 75°; monochromatic Mo *K*_α_ radiation, λ = 0.7107_3_ Å; *T ca.* 150 K) yielding 34023 total reflections, these merging to 8384 unique after empirical/multiscan absorption correction (proprietary software; *R*_int_ = 0.026), and used in the full matrix least squares refinement, refining anisotropic displacement parameter forms for the non-hydrogen atoms, hydrogen atom treatment following a riding model, 6118 with *I >* 2σ*(I)* being considered ‘observed’. Reflection weights were: (σ^2^(*F*${}_{o}^{2}$) + (0.0665*P*)^2^ + 0.482*P*)^–1^ (*P* = (*F*${}_{o}^{2}$ + 2*F*${}_{c}^{2}$)/3). *R*1 was 0.048; *wR*2 0.13; *S* 1.03; |Δρ_max_| 0.54 e Å^–3^. Neutral atom complex scattering factors were employed within the SHELXL 97 program [12]. Pertinent results are given in Table 1 and Table 2, and Figure 1a and b.

## 4. Conclusions

The facile preparation of a novel indole-substituted benzylic pyridinium salt **2** is described and the salt characterized spectroscopically and through single crystal X-ray diffraction methodology. The solid state conformation of **2** indicated an interesting overlap of the pendant pyridinium ion and the indolic 5-membered ring.

## Figures and Tables

**Table 1:** Crystal data for compound **2**.

Formula	C20H16ClN3O2
*F*w	365.8
Crystal system	Monoclinic
Space group	*P*21/*c* (#14)
*a* (Å)	10.274(3)
*b* (Å)	13.101(4)
*c* (Å)	13.439(4)
β (°)	107.702(7)
*V* (Å^3^)	1723.2(9)
*Dc* (g cm^−3^)	1.410
*Z* (f.u.)	4
μ (cm^−1^)	2.4
Specimen (mm^3^)	0.48 × 0.32 × 0.27
*‘T’*min/max	0.92

**Table 2:** Non-hydrogen interatomic distances (Å) and angles (°) for compound **2**.

Atoms	Parameter	Atoms	Parameter
**Distances**			
N(1)-C(2)	1.3859(13)	C(21)-C(22)	1.4050(16)
C(2)-C(3)	1.3710(15)	C(21)-C(26)	1.3979(15)
C(3)-C(3a)	1.4328(14)	C(22)-C(23)	1.3989(16)
C(3a)-C(4)	1.3954(15)	C(23)-C(24)	1.388(2)
C(3a)-C(7a)	1.4217(14)	C(24)-C(25)	1.381(2)
C(4)-C(5)	1.3863(14)	C(25)-C(26)	1.391(2)
C(5)-N(5)	1.4514(14)	C(22)-C(220)	1.5108(16)
C(5)-C(6)	1.4028(15)	C(220)-N(221)	1.4898(14)
C(6)-C(7)	1.3749(15)	N(221)-C(222)	1.3487(14)
C(7)-C(7a)	1.3997(14)	N(221)-C(226)	1.3478(14)
N(1)-C(7a)	1.3623(14)	C(222)-C(223)	1.374(2)
C(2)-C(21)	1.4737(15)	C(223)-C(224)	1.388(2)
C(5)-N(5)	1.4514(14)	C(224)-C(225)	1.387(2)
N(5)-O(51)	1.2309(14)	C(225)-C(226)	1.380(2)
N(5)-O(52)	1.2358(13)		
**Angles**			
C(2)-N(1)-C(7a)	108.95(9)	C(2)-C(21)-C(22)	121.53(10)
N(1)-C(2)-C(3)	109.46(9)	C(2)-C(21)-C(26)	119.37(10)
N(1)-C(2)-C(21)	120.41(9)	C(22)-C(21)-C(26)	119.10(10)
C(3)-C(2)-C(21)	130.02(9)	C(21)-C(22)-C(23)	118.93(10)
C(2)-C(3)-C(3a)	107.05(9)	C(21)-C(22)-C(220)	123.79(9)
C(3)-C(3a)-C(4)	134.46(9)	C(23)-C(22)-C(220)	117.24(10)
C(3)-C(3a)-C(7a)	106.38(9)	C(22)-C(23)-C(24)	121.32(11)
C(4)-C(3a)-C(7a)	119.03(9)	C(23)-C(24)-C(25)	119.65(11)
C(3a)-C(4)-C(5)	116.93(9)	C(24)-C(25)-C(26)	119.91(11)
C(4)-C(5)-C(6)	124.14(10)	C(21)-C(26)-C(25)	121.01(11)
C(4)-C(5)-N(5)	118.52(9)	C(22)-C(220)-N(221)	114.44(9)
C(6)-C(5)-N(5)	117.33(9)	C(220)-N(221)-C(222)	119.21(9)
C(5)-C(6)-C(7)	119.35(9)	C(220)-N(221)-C(226)	119.58(9)
C(6)-C(7)-C(7a)	117.69(9)	C(222)-N(221)-C(226)	121.17(9)
C(7)-C(7a)-N(1)	129.17(9)	N(221)-C(222)-C(223)	120.53(11)
C(7)-C(7a)-C(3a)	122.70(9)	C(222)-C(223)-C(224)	119.32(12)
N(1)-C(7a)-C(3a)	108.11(9)	C(223)-C(224)-C(225)	119.41(12)
C(5)-N(5)-O(51)	119.16(9)	C(224)-C(225)-C(226)	119.31(11)
C(5)-N(5)-O(52)	118.41(10)	N(221)-C(226)-C(225)	120.26(11)
O(51)-N(5)-O(52)	122.44(10)		
**Torsion angles (atoms denoted by number only, *N* italicized)**					
*1*-2-21-22	−63.5(2)	21-22-220-*221*	−45.6(2)
2-21-22-220	1.0(2)	22-220-*221*-226	−53.3(1)

## Supporting Information Available

CCDC-816104 contains the supplementary crystallographic data for this paper (excluding structure factor amplitudes). These data can be obtained free of charge via www.ccdc.cam.ac.uk/conts/retrieving.html (or from the CCDC, 12 Union Road, Cambridge CB2 1EZ, UK; Fax: +44 1223336033; E-Mail:deposit@ccdc.cam.ac.uk).
